# Medico-Legal Trial.—Unlawful Detention in a Lunatic Asylum

**Published:** 1859-10-01

**Authors:** 


### MEDICO-LEGAL TRIAL.—UNLAWFUL DETENTION IN A LUNATIC ASYLUM.

### COURT OF QUEEN'S BENCH, Westminster, June 21st.

(Sitting at Nisi Prius, before Mr. Justice Hill and Special Juries.)

### Ruck *v.* Stilwell and Another.

Mr. Edwin James, Q.C., Mr. Serjeant Petersdorff, and Mr. Gordon Allen, appeared for the plaintiff; and Mr. M. Chambers, Q.C., Mr. Serjeant Ballantine, and Mr. Honyman for the defendants.

Mr. Gordon Allan opened the pleadings. The first count was for assaulting and imprisoning the plaintiff in a house called Moorcroft-house, in the parish of Hillingdon, and detaining him there among lunatics and persons of unsound mind for a period of ten months. The second count alleged that the defendants were the keepers of a house licensed for the reception of lunatics, and received the plaintiff upon an order signed by one Mary Ann Ruck, as a private patient and with two medical certificates; that while the defendants had such charge of this plaintiff he recovered, which the defendants well knew, and it was thereupon their duty to transmit notice of such recovery to the said Mary Ann Ruck, but that they neglected to do so, and they also wilfully neglected to transmit notice of his recovery to the Commissioners of Lunacy, but under colour and pretence of the said order and certificates, kept the plaintiff in custody for ten months, &c. To these two counts the defendants pleaded several pleas, denying all the material allegations.

Mr. Edwin James, in opening the case to the jury, said this was an action of trespass brought by the plaintiff, Mr. Lawrence Ruck, against Dr. Stilwell, the proprietor of a private asylum for the reception of lunatics, at Moorcroft-house, in this county, and the questions to be submitted to the jury would be found of the greatest public importance. The plaintiff had been confined in the defendant's house from the 5th of November, 1857, down to the 27th of August, 1858, when he was discharged: and the questions would be as to the legal right of the defendant to detain him, and the validity of the medical certificates, which he should show were in direct violation of the Act of Parliament. With regard to the moral right of the defendant to detain the plaintiff, the jury would be satisfied beyond all doubt that the parties were not justified in confining him, and that the plaintiff required no medical treatment at all, and but merely a preventive treatment. The jury would find that the plaintiff was of sound mind, and that there was no justification for confining him either in law or morality. The plaintiff was the owner of some property in Kent and Wales, and had been educated at Oxford, and he amused himself with agricultural pursuits and the sports of the field. In the month of August, 1857, he went down to Manchester with his wife to see the Exhibition, and there met a Mr. Barnett, who was one of the gentlemen who signed the medical certificate. After staying at Manchester some little time he went to Wales, and there attended a meeting of the Newtown and Machynlleth Railway Company, and continued in Wales till the month of October. In consequence of some domestic circumstances, the plaintiff then gave way to great intoxication, and his mind became affected, as always happened more or less in such cases. He then went to Welchpool, and met with Mr. Barnett, one of the gentlemen who signed the certificate, and though it was well known to everybody that the plaintiff was not in a state of insanity, Mr. Barnett took him to Reading where his wife was. There had been domestic differences, but he (Mr. James) made no charge against Mrs. Ruck, who had acted on the advice of Barnett and his attorney. There was no pretence whatever for charging the plaintiff with insanity, and yet, even after a jury had found that he was of sound mind, the defendant went home and made an entry in his book to the effect that, in his, the defendant's judgment, the plaintiff was still "a dangerous lunatic," though it would be proved that he never once raised his hand against any one. His wife had slept with him at Reading the night previous to the day when Mr. Barnett and Dr. Conolly signed the medical certificates. The learned counsel referred to the enormous sums which were sometimes paid to the keepers of private lunatic asylums, 300*l.*, 400*l.*, 500*l.* a-year, and, in one instance, as much as 2000*l.* a-year. This was a great temptation, and therefore the legislature had interfered for the protection of the public, by passing several enactments, one of which was 16 and 17 Victoria, cap. 96. The 4th section of that statute enacted that no person should be received as a lunatic into any licensed house without an order signed as directed, nor without two certificates, signed by two persons, each of whom should be a physician, surgeon, or apothecary, who had respectively examined the person to whom the certificate related; and by the 36th section, the physician, surgeon, or apothecary must be one who was in actual practice. It would be shown that Barnett had not been in practice for years, and that both Dr. Conolly and the defendant (Dr. Stilwell) knew that fact. His certificate, therefore, had no legal weight at all, and the defendant (Dr. Stilwell), in receiving a patient on such a certificate, was guilty of a misdemeanour. The other certificate was signed by a gentleman, for whom he (Mr. James) personally had great respect, and in whom the public had great confidence, but he was astonished to find, that with the knowledge of the Act of Parliament, Dr. Conolly should have put his hand to a certificate which confined the plaintiff in a lunatic asylum for ten months. Very large sums were paid to private lunatic asylums for the board of lunatics, and therefore they required very great protection; for the interest of the keepers of those asylums was not to cure their patients but to keep them there. The defendant received as much as 400*l.* a-year for the plaintiff's board, and, as he had before stated, in one instance, as much as 2000*l.* a-year had been paid. The 12th section of the statute enacted that, "no physician, surgeon, or apothecary who, or whose father, brother, son, partner, or assistant, is wholly or partly the proprietor of, or a regular professional attendant in, a licensed house or hospital, shall sign a certificate for the reception of a patient into such house or hospital," &c. Notwithstanding that enactment, he (Mr. James) regretted to say Dr. Conolly, who had signed one of these certificates, was actually a partner in the defendant's establishment. When the plaintiff was sent to the defendant's asylum it was found that he did not require any medical attention whatever. This fact soon transpired, and also that Dr. Conolly was in the habit of giving certificates in direct violation of the Act of Parliament. Dr. Conolly received 15 per cent. of the profits of the establishment, and he had actually received his percentage of the profit of the detention of the plaintiff. The learned counsel here read an extract from the defendant's book, from which it appeared that in March, 1858, Dr. Conolly had received a sum of 15*l.* in respect of Mr. Ruck (the plaintiff). Dr. Conolly was to receive 60*l.* a-year out of Mr. Ruck's payments. He was the medical attendant at the asylum, and received as his salary 15 per cent. on the payments of all confined in the asylum. Dr. Stilwell had brought an action against the plaintiff to recover a sum of 250*l.* for his board, but as the plaintiff had been confined there against his will, he refused to pay. The certificates were not worth the paper on which they were written, and nobody knew that better than Dr. Stilwell, who had about forty-five patients in his house at the time, upon whose payments he was paying 15 per cent. to Dr. Conolly. A more scandalous violation of an Act of Parliament there could not be, and this was well known to both Dr. Stilwell and Dr. Conolly. If those facts had not been brought before the public eye, the plaintiff would have still been in confinement, and he (Mr. James) trusted the jury would express their opinion by vindicating the plaintiff for the imprisonment which he had suffered during ten months without a shadow of justice or of law. The plaintiff ought to have been discharged within a few days of his admission, but in consequence of his detention he had been put to an expense of 1100*l.* in procuring his release.

Mr. Serjeant Petersdorff then called the plaintiff, Mr. Lawrence Ruck, whose examination lasted the rest of the day—about four hours. The plaintiff, who is a strong, healthy-looking man, gave his evidence in a very calm and deliberate manner. We give only a condensed account of the evidence.

The plaintiff said he was a gentleman of independent fortune, having estates in Kent and Wales, worth about 1500*l.* a-year. He was a member of Magdalen College, Oxford, and left in 1840. In 1841 he married the daughter of a Mrs. Matthews, and by her had six children. He resided in Merionethshire, where he had two residences, eight miles apart. From about two years after his marriage, he and his wife lived very unhappily together. In August, 1857, he went from Wales to Manchester, accompanied by his wife and two daughters, to see the Exhibition, and there met Mr. and Mrs. Barnett. Mr. Barnett was formerly a surgeon at Stourport. He was a friend of Mrs. Ruck's. While the parties were at Manchester, he and his wife were several evenings with Mr. and Mrs. Barnett. He (Mr. Ruck) then went with his wife and daughters into Kent to visit his brother-in-law, and remained there two days, and then left for Wales to attend a meeting of the Newtown and Machynlleth Railway Company, leaving his wife and daughters in Kent. After taking part in the railway meeting he returned to Kent in September, and remained about a month, going out shooting, and mixing in society. He then returned to Wales to attend another railway meeting, and was re-elected a director in October. He then went to his house at Pantllwdw, where he was waited upon by a Welshman and his wife, neither of whom could speak English, he being equally ignorant of Welsh. His home was uncomfortable, and a Miss Jones was sent for by Mrs. Matthews, his wife's mother. An intimacy had previously subsisted between him and Miss Jones which resulted in the birth of two children, a fact which he was anxious to keep secret from his wife. He was exceedingly annoyed at Miss Jones's conduct, who came in a half-drunken state, and afterwards made too free with the bottled ale, while he was out. He and Miss Jones occasionally had quarrels. As the weather was unfavourable to farming pursuits, he used to go out driving, and stopped here and there to refresh himself as well as his horse, and in this way he sometimes took more than was necessary. His trouble of mind affected him as much or more than the drink, and he became exceedingly irritable. He was at Pantllwdw for a fortnight. On one occasion he found that some one (whom he surmised to be Miss Jones) had entered his gunroom and disturbed some medical books with plates, and, as he thought they were not fit to be seen by women, he burned the books in the fire. He was intending to go to Kent, and in October drove towards Shrewsbury, to a place called Carno, and though he had had no intimation that his wife was coming to Wales, he was surprised to see her drive up there. He supposed she had been home and followed him. He said he could manage himself very well, and proceeded on to Welchpool, where he had a dispute with his wife about a gentleman going inside the coach. They stayed at Welchpool three days, and while there, he and his wife occupied separate rooms. To a moderate extent he indulged at Welchpool, and at night sent for a Dr. Harrison, who prescribed for him, and he gave up stimulants. Mr. Barnett was telegraphed for he believed by his wife, and came, and they all three took the mail for Shrewsbury, and went on to Birmingham that night. He (Mr. Ruck) there slept with his wife. He had told Mr. Barnett he was going to Kent, and Mr. Barnett said he had better call on him at Reading, as he had not seen his (Barnett's) new house. He accordingly went, and arrived there at two o'clock on the afternoon of Sunday, the 1st of November, 1857. On their arrival there, Mr. Barnett and witness's wife were for about an hour in conversation. Witness settled the expenses of the journey with Mr. Barnett, and then went to dinner with his wife and Mr. and Mrs. Barnett. After dinner Barnett pushed the wine towards him, and invited him to take some, and he took a glass, and on his pressing him again, he took another half glass, but no more, as he fancied the wine was drugged. He determined to go to Kent, and started for that purpose, but was followed by Barnett, who seized him by the collar, and asked him what he meant by leaving his house in that manner. A scuffle ensued, people came up, and he (Mr. Ruck) explained to the police the nature of the assault, and that he believed Mr. Barnett had drugged his wine. As it was Sunday the policeman said there could be no charge made that day, and he would go back with him and taste the wine. This they did, and he (Ruck) drank a couple of glasses more. (Laughter.) During that and three successive nights he occupied the same room with his wife at Mr. Barnett's house. Dr. Conolly came down on the Wednesday, was introduced by Mr. Barnett as a particular friend, and talked with him (Mr. Ruck) on various subjects, and asked some questions about some quarries, and as to his wife. He (Dr. Conolly) was there about ten minutes, and Mr. Barnett was present the whole of the time. There was no plate on Mr. Barnett's door, and no signs of a practice. After Dr. Conolly had left, Mr. Barnett returned to the drawing-room, and said he thought he (Mr. Ruck) had better go and visit Dr. Stilwell. He (Mr. Ruck) at that time knew nothing of Dr. Stilwell, nor of his keeping an asylum, but he left Reading in company with one of Dr. Stilwell's servants, unconscious that he was going to an asylum. He went to Moorcroft House, near Uxbridge, and was introduced to Dr. Stilwell and his assistant, Mr. Weller. His keys were taken from him, his boxes searched, and the door locked, and he then knew in what sort of place he was. On inquiry he was informed by Dr. Stilwell that he was authorized to detain him by some medical certificates. He was never examined by any other medical gentleman besides Dr. Stilwell, but that gentleman was excessively curious about some reports he had heard respecting him. There was no examination as to his health, and he received no medicine and no medical treatment. A man named Randall attended upon him, and at first slept in his bedroom for about six months. He was never allowed to go out without a keeper. There were about forty patients in the asylum. When he had been there two or three days, Dr. Stilwell asked him to go out for a drive, which he did, and occasionally changed seats with him, while he (Mr. Ruck) sat on the box and drove the carriage. He was told that he was not allowed to post letters, and on one occasion was prevented by a keeper when he attempted to put a letter in the box. The Lunacy Commissioners attended occasionally at the asylum, but Dr. Stilwell was present all the time. The Commissioners put questions as to reports against his (witness's) wife. He was visited by his brother and sister, but they were seldom allowed to see him privately. He wished to see Mr. W. Ackworth, his attorney, and sent a letter to him surreptitiously by an attendant, for which he believed the servant was dismissed. Afterwards Mr. Ackworth came, but he was not allowed to see him except in the presence of Mr. Weller, so that he could do no more than answer the questions which he put. He (Ackworth) said he would call again in a few days, but for some unaccountable reason he saw no more of him. He wrote to his brother-in-law Mr. Fisher, the barrister, and instructed him to do everything in his power to get him out. Mr. Fisher informed him he had put the matter in Mr. Wainwright's hands, and Mr. Wainwright called upon him in April, and he (Mr. Ruck) gave him instructions to do what was necessary to procure his discharge. He (Mr. Ruck) requested Mr. Wainwright to go to Wales and make some inquiries, which he did, and communicated to him the result. Mr. Wainwright's report lulled his suspicions and dispelled all his (Mr. Ruck's) notions. He (Mr. Ruck) had to give Mr. Wainwright an order for some money, but Dr. Stilwell interfered and prevented it. He (Mr. Ruck) was not attended by any medical man while there, but during Dr. Stilwell's illness an uncle of his attended for two or three months. Dr. Conolly used to visit the asylum once a week or once a fortnight. Mr. Ruck said he was present during the whole of the inquisition, and was examined by the Master in Lunacy, in presence of the jury, some of whom put questions. The result was that the jury found he was not insane. Since August last he had resided with his brother-in-law and at home. His income was about 1500*l.* a year, and he had some quarries, respecting which he had consulted some mining agents. Upon one quarry he had expended 500*l.* or 600*l.*, and some more money on another. At Moorcroft 3*l.* or 4*l.* were given to him at a time.

The plaintiff was then subjected to a very long and rigid cross-examination by Mr. Chambers. The subject of the cross-examination was as to the plaintiff's habits of intoxication and various delusions under which it was suggested he was labouring at the time he was sent to the asylum, particularly delusions as to his wife's infidelity. He admitted that at the time he entertained those delusions, but stated that his notions on that subject were entirely removed by the result of Mr. Wainwright's inquiries.

Mr. Ruck said—Mr. Wainwright's report dispelled all my suspicions and notions. That was a month or six weeks before the commission. At the commission I was examined by the Master in Lunacy. Previous to entering Moorcroft-house I had suspicions respecting my wife's infidelity. I communicated them to Dr. Stilwell. Mr. Wainwright's report entirely removed my suspicions. All my suspicions have now been removed. My wife has only had 40*l.* or 50*l.* from me, but I consider she has had enough from the sale of stock. She has six children to support. She sold the stock on my farm for 1500*l.* There was an agreement that my wife was to have 10*l.* a-week, and to sign a deed of separation. The deed was prepared, but she has hitherto refused to sign it. On one occasion she said she had not a farthing in her pocket, and Mr. Wainwright gave her a cheque for 20*l.* My first suspicions of my wife were at Carno. A gentleman got into the coach, and I thought my wife and he were too intimate, and I refused to go any further. At Welchpool I suspected her with regard to some of the people in the hotel, but no one in particular. I heard noises and footsteps at night outside my door, and thought my wife might be going about the house in a loose kind of way. Two men were watching. I was in a very agitated state. We had lived unhappily together after the first twelve months. I had a house at Pantllwdw, and another eight miles off, at Aberdovery, where my children lived. I never fancied people were poisoning me. I did not fancy that at Lloyd's, the inn at Welchpool. I may have poured the wine into the chamber-pot. (Laughter.) It was execrable stuff. (Laughter.) I never said I had been poisoned. I had taken a fly at nine o'clock in the morning, and got into the carriage at the hotel door. I did not harness the horse myself. I took a traveller's coat for my own. It was exactly like my own. I returned it. I may have been drinking in the bar, but I was conscious when I went to bed, and when I left in the morning. The fly took me to Carno, and my wife came there. She was in an excited state. I was with my portmanteau, and going to Kent. My wife was at first unwilling to accompany me, but after a time she was willing. I did not on the journey say that I saw people rushing into one another's arms. I said that I overheard some one asking whether, when we met, we did not rush into one another's arms. I ordered a fly to take my wife back to her mother's, as I did not consider her in a fit state to travel. I suspected she was too intimate with the gentleman in the coach. When they brought the fly round my wife prevented them from going. I waited for some hours, and they said they would bring some horses; and for about a quarter of an hour I took my seat in a fly. At night I found I could not sleep, and I sent for Mr. Harrison. It was two nights after that that the two men were set to watch me. I told Mr. Harrison my wife had been intimate with a gentleman—not with most of the people in the inn. I may have had an impression that Mary Jones lay by my side, and that she was talking to me, on the first night I was at Welchpool. My head was full of noises, and I thought I heard her whispering. That was one of the notions that Mr. Wainwright removed. He satisfied me that she (Mary Jones) was twelve miles off at the time, at Dolgelly. Mr. Harrison saw me again the next day, and I had some cooling draughts. I had my gun-case and pistol-case with me. I was going a-shooting, and my pistol required repairing. I am not sure whether the pistol was loaded. I always kept my gun and pistols loaded. The house at Pantllwdw was damp. I did not open the case. Mr. Barnett came down on Saturday after. I travelled that day. I told my wife to give me her money, and she gave me her purse with a 20*l*.-note in it. I did not know the 20*l*. was a present from her mother. We arrived at Birmingham at between twelve and one o'clock in the morning, and we started at ten o'clock the same morning. I have no recollection of saying to my wife, "You may well say your prayers." I did not go to my gun and pistols. I suppose that must be my wife's evidence. I may have said, "We must make haste and get away from here, for they know what you are." I suspected that my wife had been familiar with some people or other. I account for this by the fact that the noises in my head continued; and under certain conditions you may imagine almost anything. I did not say it had been telegraphed north, south, east, and west. No doubt I had the noises in my head on the way to Reading. They continued several days. I thought my wife had disgraced me with some men at Welchpool, and I said I would abandon the county. (The witness was here cross-examined at great length on these matters, and admitted the saying of many things against his wife which he was now satisfied were unfounded.)

The jury interposed, and asked whether it was necessary to go into these matters so fully, as they were admitted.

Mr. Justice Hill said he could not say that the evidence was irrelevant.

The plaintiff continued.—At the police-court, at Reading, I said my wife was nothing but a ——. That was a day or two before I went to Dr. Stilwell's. When Miss Jones came to me at Pantllwdw she refused to tell me what she had done with her children. I did not send for a policeman to give her in charge for murdering her children. I locked her up. She was in that drunken state that she was better locked up. (Laughter.) My wife never contradicted me, or appeared to feel injured, when I spoke of Mary Jones. At Reading I kept quiet, and ceased to have noises. I have not had noises in my head during the last six weeks; not at Sittingbourne, where I was living with a job-master. At Reading I dined with Mr. Barnett. I may have taken a glass or two of sherry at dinner. I had got half-a-mile when Mr. Barnett came after me. We struggled, and I got him on the ground. If I had been inclined, I could have given him such a settler as would have stopped all further inquiry. (Laughter.) He certainly had no right to follow me and insult me as he did. I told the policeman my wine had been drugged. He said that was a serious charge, for drugs were poisons. I said the wine was not poisoned, but drugged. We all went to the station, and there I told the inspector my wife was a ——. I said no doubt he (Barnett) wanted to lay me up by drugging me, because I said my wife was a ——. It was to prevent me from leaving my wife. I suspected that Barnett was one of her paramours. I did not mention that at the police-station. I told Dr. Stilwell so soon after my arrival at Moorcroft. Being detained in that house strengthened my suspicion that I was detained there to save my wife from exposure. I said to the doctors I had no reason to disbelieve my suspicions, because I had no means of making inquiries. As soon as I received Mr. Wainwright's proof positive that all my suspicions were groundless I was satisfied. I have no doubt that my wife has been a pure and faithful wife all her life. I have no doubt of it. I have expressed great contrition to her. I thought all was condoned when I slept with her three nights at Reading. In May or June, 1858, I did not say that Mr. Barnett and my wife had had illicit intercourse. I may have said to Dr. Sutherland, in July, 1858, that all the servants on the railway took marked notice of my wife, but not that they said, "That is the woman that bilked her husband." I did not tell Dr. Winslow, in July, that no London prostitute could conduct herself as bad. I never said my wife attempted to poison me. I may have said she drugged my wine. I may have said that to Dr. Winslow at one of his visits. Mr. and Mrs. Goord did not come to see me at the asylum. Mr. Fisher and my sister came in November. I have no doubt I told him to do all he could to get me out. I did not see Mr. Wainwright till the spring, in April. The commissioners spoke to me from time to time, but in the presence of Dr. Stilwell. I never asked to see my children; I did not wish them to know I was in such a place. It was through Mr. Fisher Mr. Wainwright was employed. Mr. Wainwright got an order to see me, and he then saw me, without interruption, four or five times. Mr. Williams was introduced to me as a friend, but he betrayed all my confidence. I was allowed to go to London to see medical men, and I once dined with Mr. Fisher. Going home we had some brandy and soda-water and cigars, which had some effect on me; but when I got home I lighted my candle, and walked upstairs, and went to bed, and put my candle out. In about five minutes a man came into my room, and I called out, "You fancy you are going to catch a weasel asleep." (Laughter.)

June 22nd.

Mr. Ruck, the plaintiff, was recalled, and his cross-examination resumed. He said:—Miss Jones is a cousin of my wife's, who often visited at my house; but my wife did not know that I had had two children by her. I did not say at the commission that she had murdered her children. She would not give me any information respecting the children, and I thought she might have made away with them. I now know from Miss Jones that they are well taken care of. She behaved most shamefully to me, and, as she has ample means of her own, I have almost disowned the children. She is dependent on her uncle and aunt. I once sent her a 10*l*.-note by a friend. A short time ago she asked me for 500*l*., and I thought it so ridiculous that I took no notice of it. When I saw Mr. Wainwright, he told me I had better see no one without a letter of introduction from him. I was on my guard in talking to strangers. I am on my guard now. (Laughter.) The unhappiness which took place after my marriage was from my wife refusing to have any connexion with me. She did that for five years. We were very cool to one another. I always acted towards her in a proper manner. She treated me in the same style. At Reading, I and my wife condoned one another's conduct. I have mortgaged my property to a considerable amount to pay the law expenses.

Re-examined by Mr. Serjeant Petersdorff.—In respect to the quarries, I acted on advice. There never was any insanity in my family. This entry in the book produced was written the morning after I went to Welshpool. I was very unwell at the time. My portmanteau was taken from me at Reading, and all my books, including this one, were returned to my wife. I first became acquainted with Miss Jones some years ago. She used to visit in the house for months. It was Mrs. Matthews, my wife's mother, who sent for Miss Jones to my house. I can't say that Mrs. Matthews knew of the intimacy which had taken place, but I surmised ——. I never saw Miss Jones's two children. Miss Jones's father was a man of some property. He was a captain in the navy. I had a great quarrel with Miss Jones when I was in Wales. From the time I went to the asylum till the time I was discharged Dr. Stilwell never attempted to remove my delusions. I saw him almost daily. He ceased to talk about my delusions after five or six days. He never endeavoured to remove my delusions. I believe he inquired of my attendant, but not of me. I never had any noises in my head till October, when I went down to Wales. When I condoned the matter at Reading, I said I should think no more of it, and we had better go into Kent and live together. She agreed to it. That was on the Tuesday, before Dr. Conolly came. When I left the asylum I found that property had been sold for 1500*l.* which I valued at about 2000*l.* The house at Pantllwdw was let. I was at Aberdovey when I heard my wife say she had not executed the deed of separation, and that she wished to hear from her sister. About three or four years before the commission my wife had said we had better part. Sometimes we lived at different houses. She lived at Aberdovey and I at Pantllwdw for about two years; but I visited backwards and forwards. I did not want my wife to come with me from Wales in October, because she had no luggage.

George Randall, examined by Mr. Allan, said:—On the 5th of November, 1857, I was an attendant at Dr. Stilwell's asylum, and took charge of Mr. Ruck. My orders from Dr. Stilwell were never to allow him to post letters, and never to lose sight of him. About two days after Mr. Ruck came he went out for a drive with Dr. Stilwell. He was then as quiet as any gentleman need be in the world. He told me he drove. I had care of him for four months, and went out to walk with him. He went to church, and always conducted himself like the rest of the gentlemen there. I accompanied him to Windsor on several occasions. He had some money (3*l.*), and my orders from Dr. Stilwell were to let him spend it as fast as he could. He spent it very frugally. 3*l.* lasted about a month. He never had any medicine while I was there. When Mr. Ruck had been there three weeks, Dr. Stilwell told me it was very strange he could see nothing the matter with him. Dr. Stilwell told me to report to him about Mr. Ruck, and I used to report that I could see nothing the matter with him. When Mr. Ruck had been there about a month, Dr. Stilwell said to me he could see nothing the matter, and he would soon be discharged. He said that to me on several occasions; I might say half-a-dozen times. There were two physicians to the establishment, Dr. James Stilwell, of Uxbridge, and Dr. Conolly. They attended there once or twice a-week regularly during the whole time I was there. Dr. Conolly used to visit the patients. If Dr. Stilwell (the defendant) was not at home, Mr. Weller used to take Dr. Conolly round the rooms. I had been at Moorcroft about a week before the 5th of November, and I had seen Dr. Conolly there before that date.

Cross-examined by Mr. Chambers.—I saw Dr. Conolly enter at the street-door, and saw him go to the patients. He shook hands with the patients, and asked them how they were. The grounds were half-a-mile round. Dr. Conolly several times talked to Mr. Ruck. I should say seven or eight times. There was a ball there, and Dr. Conolly was at the ball. I left the establishment of my own accord, about a month after Christmas, I believe. I was there about four months. I recollect Mr. Weller being drunk one night. He bullyragged me. (Laughter.) When I was before the Master in Lunacy I said nothing about Dr. Stilwell's telling me to let Mr. Ruck spend the money as fast as he could. The question was never asked. But I say it now. (Laughter.) Mr. Ruck talked to me about his suspicions as to his wife up to the time I left. I told him they were delusions, I should say; he must be mistaken in his notions. I might have told him that once or twice. I was to report to Dr. Stilwell if Mr. Ruck misconducted himself out. Dr. Stilwell asked me if Mr. Ruck talked to me about his wife and Mr. Barnett. I told him once or twice that he did, but I did not see anything the matter with him. I told Dr. Stilwell Mr. Ruck had said Mr. Barnett and his wife had been too intimate, too familiar. That was one of the delusions in which I told Mr. Ruck he must be mistaken. Another was that he said he had seen his wife nod and laugh with a traveller in a coach in Wales. He did not say his wife had had connexion with a traveller who got into the coach. He said Mrs. Ruck paid too much attention to him, and they were too familiar. Mrs. Ruck came to Moorcroft, and after that I told Mr. Ruck that he was mistaken. Mr. Ruck did not tell me he suspected his wife had had connexion with men in the inn at Welshpool. He might; I have no recollection. After I saw Mrs. Ruck I told Mr. Ruck his wife was a very ladylike person, and he must be mistaken about Mr. Barnett and the man in the coach. That was after Mrs. Ruck had had an interview with Mr. Ruck. When I reported to Dr. Stilwell, he used to say he could see nothing the matter with Mr. Ruck. I said at the commission that he said it on one occasion, but I am sure he said it more than once. My memory has got better now. I believe Mr. Ruck was as sane as anybody in court all the time he was at the asylum. He told me he had been drinking, and I explained to him that he might be mistaken. I posted a letter for Mr. Ruck. It was some time after I left. When I left I complained of Mr. Weller's language, and said I would "open the ball upon the asylum." I meant I should go and see Mr. Ruck's friends, and just tell them I could see nothing the matter with him. I saw Mr. Ackworth.

Re-examined by Mr. James.—I discharged myself. Mr. Weller had a party, a regular flare-up. We would not join him, and remained in the kitchen. The next morning I left. Mr. Weller was drunk on that occasion, and on several other occasions. Mr Ruck was always very quiet. He was never a dangerous lunatic. He never raised his hand. He told me he used to take brandy about with him, and take too much, and he did not know what he was about. He said he courted inquiry, but he was shut up there.

By a Juror.—When I went out with Mr. Ruck we had half-and-half, but no spirits. Wine was allowed in the establishment.

Thomas Randall, examined by Mr. James, said—I am now in the police force. I am the brother of the last witness. I was a keeper in Moorcroft-house from three weeks after Mr. Ruck came till the 12th of May, 1858. I used to walk with the plaintiff, and never observed anything like insanity about him. He was not violent. I received orders from Dr. Stilwell not to post any letters. On the 9th of May, Dr. Stilwell said to me, "Have you been posting a letter for Mr. Ruck?" I said, "No." He turned round with a vicious look and said "You have." He said, "You have; the lawyers are at work, and no doubt will get him out." I was discharged for posting the letter. I had a month's money. My brother posted the letter, and I would not tell. I was discharged innocently. I have seen Dr. Conolly going round the rooms to the patients twice a-week. Dr. Conolly attended a patient of whom I had care.

Cross-examined by Mr. Chambers.—Dr. Stilwell had an uncle, Dr. James Stilwell, who lived at Uxbridge. He did not attend almost every day. I have seen him there once, twice, or three times a-week. I thought Mr. Ruck was sane. I did not speak to him about any delusions.

Theodore Prichard, examined by Mr. Serjeant Petersdorff, said—I am now in a regiment of Hussars. I was a keeper at Moorcroft for eight months, and left in August, 1858. I saw Mr. Ruck every day, and used to accompany him in his walks in the meadow about once in the week. I was in the habit of talking to him. I thought he was quite sane. I never observed anything insane in him. Dr. Stilwell gave me orders not to post any letters for Mr. Ruck. I have seen Dr. Conolly at the asylum. He has come into the room where I

was sitting with the patients, and asked how they were. There were five or six patients in the room at the time. I have seen him once a-week, once a-fortnight, or three times a-month. He felt the pulses of the patients. I have seen Dr. Conolly in different rooms with patients.

Cross-examined by Mr. Serjeant Ballantine.—I was there for eight months. Mr. Ruck was the same all the time. The rule was, that letters from the patients were to be put on the hall table. Dr. Conolly came occasionally. Dr. James Stilwell came two or three times a-week. Dr. Conolly went through the house with Dr. Stilwell, and on some occasions with Mr. Weller. I was not much with Mr. Ruck. I have talked with him about different things, but never about his wife or family.

R. D. Jones, examined by Mr. Allan, said:—I am a magistrate, and reside in Montgomery. I knew the plaintiff, Mr. Ruck, but we were not intimate. I was a director of the Newtown and Machynlleth Railway Company, and met Mr. Ruck, who was also a Director, in October, 1857. He attended several meetings in the autumn of that year, and took part in the proceedings. What he said was very sensible and to the purpose. There was nothing to show that he was a lunatic. No remark was made by any of the directors of any singularity. I have heard that Mr. Ruck had a slate quarry, and have talked to him about it.

Cross-examined by Mr. Serjeant Ballantine.—I never had any conversation with Mr. Ruck respecting his wife till after his release. I interfered to procure a reconciliation between Mr. Ruck and his wife, but failed.

The medical certificates and order for the admission of Mr. Ruck into Moorcroft Asylum were here put in and read.

Dr. Conolly's certificate, dated the 3rd of November, 1857, stated that he had examined the plaintiff, Lawrence Ruck, at Reading, on that day, "separately from any other medical practitioner," and that he was "a person of unsound mind," &c., and he formed that opinion on the following grounds:—

"1. Facts indicating insanity observed by myself.—A disposition to talk openly of his having had ocular proof of the infidelity of his wife, and speaking of it as if it would not interfere with their future comfort; and an exaggerated manner of talking of the value of quarries on his estate.

"2. Other facts (if any) indicating insanity communicated to me by others.—An uncontrollable disposition at intervals to intemperance, acts of extravagance, and fits of depression, in which he has spoken of suicide. He has also the habit of carrying firearms with him. (Communicated by one of his friends, Mr. Barnett, of Reading, and by Mrs. Ruck.—J. CONOLLY, M.D., Hanwell."

Mr. Barnett's certificate, dated the 5th of November, 1857, stated that, "being in actual practice as a surgeon and general practitioner," he "separately from any other medical practitioner had examined Mr. Ruck, and that he was a person of unsound mind," &c., and that he formed that opinion on the following grounds:—

"1. Facts indicating insanity observed by myself.—Charges of cruelty against others, which are false; restlessness, and a determination to wander forth, with complete infirmity of purpose; indulging in intemperance, and a profligate expenditure of money; taxing his wife with infidelity; inconsistent in alluding both to his agricultural operations and to the value of the land, from a supposition of immeasureable amounts of mines, lead, copper, and slate, contained therein.

"2. Other facts (if any) indicating insanity communicated to me by others.—Going about with loaded pistols; threatening those who have always been in close bonds of friendship with him; building walls one day, and altering them the next; in all his actions presenting the converse of his former self; excess of intemperance, or total abstinence; embarking money injudiciously and recklessly in speculations. (Mrs. Ruck.) RICHARD BARNETT, M.R.C.S."

"11, Victoria-square, Reading."

The order for the plaintiff's reception by Dr. Stilwell, at Moorcroft-house, signed by Mrs. Ruck, was also read. It was dated the 5th of November, and stated that the patient, Lawrence Ruck, was thirty-seven years of age, married, a landed proprietor and farmer; a Protestant of the Church of England, living at Pantillwdw, in Merionethshire, North Wales; that the present was his first attack, now extending over two or more years; that he had not been under any treatment previously; that the cause of his attack was probably partly hereditary and partly from intemperance, and that he was not subject to epilepsy. To the question, "Whether suicidal" the answer was, "No, I think not;" and to the question "Whether dangerous to others?" the answer was, "Has carried pistols loaded, and threatened others." It also stated that he had not been found lunatic by inquisition, and that there were no special circumstances preventing the patient being examined before admission, separately, by two medical practitioners.

Mr. James then called for extracts from one of Dr. Stilwell's books, headed "Dr. Conolly." They were produced, and read thus:—

"Mem. for Dr. Conolly, for
Quarter ending the 30th of March, 1858.
February 5, Ruck . . . . . £15 0 0
Mem. for Dr. Conolly, for
Quarter ending June, 1858.
May 5, 1858, Ruck . . . . . Qy. £15 0 0"

Mr. James submitted that the whole book was evidence, including the part which was sealed up. The question was, whether Dr. Conolly was wholly or partly a proprietor of the house.

Mr. Serjeant Ballantine objected that the book was not evidence, except as far as it was produced under the judge's order. The parts not ordered to be inspected had been sealed up.

Mr. Justice Hill said he had consulted with Mr. Justice Erle on this point, which was a novel one, and his opinion was that nothing was produced but the exposed part; but the plaintiff would be at liberty to call on the defendant to open the sealed part, and if the defendant refused, the plaintiff might give secondary evidence of the contents, and make observations on its not being opened to the jury.

Mr. Chambers upon that intimation consented that the book should be opened. This was done at a subsequent stage of the trial.

Mr. T. N. Walford, examined by Mr. Allan, said he was a surgeon, residing at Reading, and knew Mr. Barnett, of Victoria-crescent, Reading. In 1856, Mr. Barnett called upon him, and he returned the call, and had met him since in the town and at the Pathological Society, but never met him in practice.

Mr. G. W. C. Wainwright, examined by Mr. Serjeant Petersdorff, said:—I am the attorney for the plaintiff. I first went to Moorcroft-house on the 7th of November, 1857. I was applied to by some relatives. I saw Dr. Stilwell and Mr. Weller. Dr. Stilwell said Mr. Ruck had been suffering from *delirium tremens*, and he could not tell what effect seeing us might have; he would only allow us to see him from one of the windows. We went up to a window, and Mr. Ruck and Mr. Weller came round. That was all I saw. I saw the certificates. I went again on the 26th of April. Then I only saw Mr. Weller. I applied to see Mr. Ruck, and did see him. My impression is that on that occasion Mr. Weller was present. Mr. Fisher was present. We proposed to bring down some medical man. Mr. Weller said there was no objection, and on the 28th I took down Dr. Seymour. Mr. Ruck complained of being detained. On the 12th of May I saw Dr. Stilwell, and told him Dr. Seymour thought it was not a case for confinement in a lunatic asylum, and he considered that if Mr. Ruck were kept quiet he would soon recover. Dr. Seymour gave instructions for some confidential inquiries to be made, and he would see Mr. Ruck with the result. A conversation then took place between me and Dr. Stilwell whether Mr. Ruck was likely to be removed. Dr. Stilwell wished to know, and I said that was my view, that it would be better that he should be removed. Dr. Stilwell then pointed out that Moorcroft was a very beautiful place, that no asylum had larger grounds, and that as to attendance, he was always there, and Dr. Conolly, of Hanwell, attended. I arranged to come again on the 19th to see Mr. Ruck. Mr. Ruck then said he would have the whole matter sifted. Dr. Seymour and I had previously ascertained what those matters were, viz., the man in the coach, Miss Jones's being at Welshpool, the infidelity of his wife, and the *fracas* at Reading. I did not go again in consequence of a letter from Dr. Stilwell to Mr. Fisher. I next saw Mr. Ruck on the 25th of June. I had not then prosecuted the inquiries. On the 25th of June I was instructed to make inquiries. I was instructed on the 6th of July. I made my report on the 25th of July. On my doing so, he admitted that there was no doubt he had been wrong. On the 19th of May, I told Dr. Stilwell that I meant to get Mr. Ruck discharged, and I had no doubt of the result. Mrs. Ruck was present, and asked Dr. Stilwell if there could be any doubt of the result after what the doctors had said. Dr. Stilwell said, "Yes, there will be a great conflict of evidence, and the medical men on both sides will be to some extent biassed by the side they take." After I got the decision of the Lords Justices, I saw Dr. Stilwell, and arranged with him for Mr. Ruck to be brought up on the commission. After the commission terms were come to for a separation. The deed I believe has not been executed. The expenses of the commission were 1100*l.*

Cross-examined by Mr. Serjeant Ballantine.—Mrs. Ruck told me she never would live with Mr. Ruck again. I communicated that to Mr. Ruck. She afterwards met Mr. Ruck at my office, after the inquisition, and said there was no reason why they should not live together. Mr. Ruck declined, and referred to the former interviews, when some very unpleasant matters took place. It was proposed that a cottage should be taken for Mr. Ruck, and I suggested, in answer to Mrs. Ruck, that Dr. Seymour would be a proper person to put him under. After I had made the inquiries, I explained to Mr. Ruck the state of intoxication in which he was, and I told him what each person had said. From that time he was cured.

Re-examined by Mr. James.—I never observed any act or word of insanity in Mr. Ruck that would justify his confinement.

At this stage of the proceedings Mr. Justice Hill, who had opened and examined Dr. Stilwell's book, headed "Dr. Conolly," read the entry made in the quarter from January to March, 1857. There were the names of fifteen patients, with various sums put after each name, except two. There were five patients whose names had 6*l.* 5*s.* after them, two with 10*l.*, and six with 12*l.* 10*s.*, making a total of 126*l.* 5*s.*, to this was added "consultation fee, twenty-five guineas," thus making a total of 152*l.* 10*s.* for that quarter. The next quarter, ending Midsummer, 1857, had similar entries, in respect of eighteen patients. The quarter ending the 30th of March, 1858, had similar entries, amounting to 156*l.* 5*s.*, but no consulting fee. Opposite Mr. Ruck's name was 15*l.* The quarter ending Midsummer, 1858, had a consulting fee, 26*l.* 5*s.*, and from fifteen to eighteen patients, with various sums opposite their names, Mr. Ruck's having 15*l.* after it.

Mr. James then put in the entry made by Dr. Stilwell in his book after the close of the inquisition, as follows:—

"August 27.—Mr. Ruck was discharged by the decision of the jury on his case, the full particulars of which are fully given in the morning papers. All that is necessary for me to state is, that I still adhere to my opinion that he is a most dangerous lunatic.—G. J. S."

Mr. Wainwright.—The charge made for Mr. Ruck's board was 400*l.*

Mr. James said he wished that fact to be proved, because it showed that Dr. Conolly had 15 per cent. on Mr. Ruck's payment.

Dr. Johnson, a physician at King's College hospital, examined by Mr. James, said :—I examined Mr. Ruck on the 9th and 12th of August, 1858, and have heard the evidence. The symptoms of his disease were those of *delirium tremens* arising from intemperance. I considered him sane. I do not consider that a person in such a state required detention. Such patients usually recover speedily and do not require detention.

Cross-examined by Mr. Chambers.—The safety of letting a man out of an asylum depends on the nature of his delusions. There are in our pauper asylums many who suffer from *delirium tremens.* That sometimes passes into insanity. The delusion as to the fidelity of one's wife is attended with risk.

Re-examined by Mr. James.—Repeated attacks of *delirium tremens* sometimes pass into insanity. I would not lock a man up because he was jealous of his wife. (Laughter.)

Mr. Skey, examined by Mr. James, said:—I am one of the senior surgeons at St. Bartholomew's. I examined Mr. Ruck on the 12th of August, and he appeared to be in a sound state of mind. The delusions which arise from *delirium tremens* generally pass off. Such cases are cases for detention, but not for detention in lunatic asylums. Some cases require watching, but I decidedly object to such cases being sent to a lunatic asylum, unless there is a part of the asylum devoted to such cases. I saw nothing in Mr. Ruck to show that he was insane or dangerous.

Cross-examined by Mr. Chambers.—Some patients are better under individual observation and kept alone, and some are better when placed in an establishment, such as a lunatic asylum. If a person had *delirium tremens,* I would place him under the eye or charge of a medical man, but also under a keeper. There is danger of violence in the early stages of *delirium tremens.* Delusions arising from *delirium tremens* soon vanish and pass away. It is a great object to obtain sleep. In thirty years' experience I cannot recollect any case of *delirium tremens* going out of the hospital to a lunatic asylum. Dr. Conolly has devoted himself for years, and successfully, to the study of insanity. The country owes a great deal to Dr. Conolly. I call in Dr. Conolly myself more than any other physician.

Re-examined by Mr. James :—In my judgment, from what I saw of Mr. Ruck and heard I do not think this was a case for confinement in a lunatic asylum.

Mr. J. Gay, examined by Mr. Serjeant Petersdorff, said he was senior surgeon of the Great Northern Hospital. He saw Mr. Ruck on the 5th of August last. He gave similar evidence to the previous witnesses.

On cross-examination by Mr. Serjeant Ballantine, he said that *delirium tremens* was a state of temporary insanity produced by intemperance, that it was attended with violence and delusions, and required restraint for a time.

In re-examination by Mr. James, he said the duration of delusions depended on the impression made on the mind.

Mr. E. Canton, examined by Mr. Allan, said :—I am surgeon to Charing-cross Hospital, and examined Mr. Ruck in August last, and believed him to be in a state of sound mental health. I attributed the delusions which he had when he entered the asylum to *delirium tremens.* It was not proper treatment to put such a person in a lunatic asylum.

Cross-examined by Mr. Chambers.—I am speaking of acute cases of *delirium tremens.*

Re-examined by Mr. James.—Cases of *delirium tremens* are first brought to the hospital.

Mr. James then put in the defendant's licence, and said that was the plaintiff's case.

Mr. Chambers submitted that the order and certificates which had been put in established a defence to the first count, under the 99th section of the 8th and 9th Victoria, cap. 106, which enacted that any keeper of a licensed house receiving a patient under a "proper order" should be authorized to detain him until he died, or was removed or discharged by due authority. The order was sufficient if it was good on the face of it, and accompanied with proper certificates. The second count was framed on the 19th section of the 16th and 17th of Victoria, cap. 96, which enacted that when the patient recovered, the keeper of the asylum was bound to give notice to the person who signed the order for the admission. That was disproved by the evidence, for it appeared the defendant was always of opinion that the plaintiff was a dangerous lunatic.

Mr. Justice Hill said the plaintiff's counsel rested his case on the fact that Dr. Conolly was either the regular medical attendant at the asylum or a partner, and that the defendant knew it.

His Lordship thought there was hardly any case on the second count, but he would not call on the plaintiff to elect at present.

The trial was then adjourned, and the Court rose.

June 23rd.

When the trial of this cause was resumed,

Mr. Chambers said that he had considered last night the propriety of calling witnesses, and he had determined not to do so.

Mr. James said that, under those circumstances, he should rely on the first count only.

Mr. Justice Hill said that would narrow the issues to one single question—viz., whether Dr. Conolly was a part proprietor in the defendants' establishment. He should leave the question to the jury, telling them that, if they found that in the affirmative, the verdict must be for the plaintiff; but he should give the defendant leave to move.

Mr. James said he had also made two other points, that Mr. Barnett was not a surgeon in actual practice, and also that the medical men did not examine the plaintiff apart.

Mr. Justice Hill said there was no evidence that this was known to the defendants, but he would leave those questions to the jury.

Mr. Edwin James then summed up his evidence. He said the defendants' counsel in the exercise of his discretion had determined not to call witnesses to contradict the plaintiff's case. The reason why that case was unanswered was because it was unanswerable. Dr. Stilwell and Dr. Conolly had both been in court, and yet neither of them was called to contradict or explain the evidence which had been given. A whole array of medical names had been mentioned yesterday, but now not one of them was called to contradict the evidence of Mr. Skey and the other medical witnesses, no doubt because it was felt they might go further and fare worse. The learned counsel then stated that, by the common law, a person could not justify the confinement of another, unless he could show that he was a "dangerous lunatic;" but these asylums in modern times had been made to play the same part as the monasteries did in the Middle Ages, and persons had been confined in them for the most flagitious purposes. It was for that reason the law said no one should be confined in a lunatic asylum except on two medical certificates; but the law provided that those certificates were bad if either of them was signed by a gentleman who was either a part proprietor in the establishment or a regular medical attendant at it. And yet, in this case, it appeared that one of these certificates was signed by Dr. Conolly, who was in the receipt of nearly 800*l*. a-year from that establishment. Another fact in this case was that, in an important public investigation like this, which was interesting to every one, the Lunacy Commissioners had not come forward to justify their conduct, and show, if they could, that this gentleman, who, according to the evidence, ought never to have been in a lunatic asylum at all, was properly confined. One point which he had to show the jury was, that Dr. Conolly was a part proprietor in the defendant's establishment. That would be proved by the book, which showed that he had received 15 per cent. on Mr. Ruck's payments. That showed that Dr. Conolly had the best of it, for he shared in the profit without sharing in the risk. What was the reason why Dr. Conolly was not called? Was it not because he would have been obliged to confess that he was receiving 15 per cent. on the payments of a number of those patients who were confined on his certificates? He (Mr. James) believed, from what had occurred elsewhere, that, if Dr. Conolly had been called, he would not have denied that he was a part proprietor and a regular medical attendant at the asylum. The learned counsel made some strong observations on the neglect of duty of the Lunacy Commissioners in not coming forward to justify their conduct.

Mr. Justice Hill interposed, and observed that on this inquiry the Commissioners were gagged, and had no power to be examined, unless called by one of the parties.

Mr. James said the defendant might have called them, and the very fact that he had not done so satisfied him (Mr. James) that the Commissioners had neglected their duty. The plaintiff never was insane, and ought never to have been locked up. He was suffering from *delirium tremens*, in which it was well known persons had delusions; but why should he have been locked up? He had given the greatest proof of sanity in the witness-box by narrating all the details of what had occurred while he was suffering from the delirium. The learned counsel then referred to Mr. Barnett's certificate, and asked why Mr. Barnett was not called. If he could have shown that his certificate was true, or that he had ever been told what he had stated, that would not have been an answer to the action, but it would have gone in mitigation of damages. But there was not a word of evidence to show that one word of the certificate was true, except the plaintiff's suspicions of his wife's fidelity, and his intemperance, which were admitted. The learned counsel went through the certificate, commenting on its various charges—"a profligate expenditure of money," "going about with loaded pistols," "threatening those who have always been in the bonds of friendship with him," charges which, he said, were entirely disproved. Then he was charged with "building walls one day and altering them the next" (*diruit*, *ædificat*, *mutat quadrata rotundis*), and "embarking money injudiciously and recklessly in speculations." If such charges could prove a man to be insane, the learned counsel thought a large portion of the world might be pronounced insane, and shut up. Such, however, were the grounds on which Mr. Barnett formed his opinion, though it now appeared that, with one or two exceptions, there was no foundation for them. The learned counsel then went through Dr. Conolly's certificate, and ridiculed the notion of locking up a man in a lunatic asylum upon such grounds as were there stated. He verily believed that, if one of the "Mad doctors" were locked up for half-an-hour with the greatest man in England, he would find out that he was insane. He would find that he had an exaggerated opinion of his abilities, or an inordinate self-conceit. If the party examined were a barrister, the doctor would probably find that, though poor and briefless, he expected to be Lord Chancellor. Dr. Stilwell, the defendant, had sat in Court, and heard all the witnesses had to say against him, and yet he would not come into the box to contradict one word of it. The only defence to the action at common law would be, that the plaintiff was a dangerous lunatic; but there was no defence on that ground, for there was not one entry in the defendant's books during the ten months he was at the asylum to show that the plaintiff had ever raised his hand, or spoken an unkind word to any one. The defendants must, therefore, rely on the certificates, which were both bad; Mr. Barnett's because he was not a surgeon actually in practice, and Dr. Conolly's because he was a part proprietor of the establishment and a regular medical attendant at it. The learned counsel contended that the plaintiff's was not a case to be received into a lunatic asylum at all, and even if it was, he ought to have been received for the purpose of care, and not to be imprisoned. The learned counsel here read a letter written by Dr. Stilwell, on the 9th of June, 1858, refusing permission to the plaintiff's own brother-in-law, Mr. Fisher, to come and see him, and concluded a most effective address by calling upon the jury to mark their view of the defendants' conduct by such a verdict as would teach the keepers of such establishments that they must treat their inmates with justice and according to law. (The learned counsel's speech was followed by applause.)

Mr. M. Chambers then addressed the jury for the defendants. He said he was glad to hear those sounds of applause, for it convinced him that topics which had nothing to do with the case had made their due impression. Many of Mr. James's observations were most eloquent, but they were also most unjust towards the defendants. The law of England was strong to prevent a person from being improperly shut up in a lunatic asylum, for, if he had but humane relations to interfere in his behalf, it was as easy to procure his discharge as it would be to procure a prisoner's discharge from prison. He would undertake to show that Dr. Stilwell was not subject to the gross imputations which had been charged against him, and he would have as little difficulty in relieving Dr. Conolly from the aspersions cast upon his reputation. The law was, that no one could keep a lunatic asylum unless he was duly licensed so to do. He must apply to the Commissioners of Lunacy for a licence, which was granted only for thirteen months, and then required to be renewed. Before he could receive a patient he must have an order, accompanied with two medical certificates that the patient was of unsound mind, and those certificates must state on their face the facts on which the medical men grounded their opinion. When an order was presented, accompanied by such certificates, the keeper of an asylum was bound to take for granted all he found stated in the order and certificates. Another protection afforded was in the Lunacy Commissioners, who were bound to visit those establishments from time to time, in the discharge of their public duty, and no doubt they would faithfully discharge that duty, though Mr. James had assumed that they would not do it. A great deal had been said about "Mad doctors," which reminded him of the cry of "Mad dog!" He would ask, what dog would get justice when there was a cry of "Mad dog?" A great deal had been said about the orders given not to post letters, the regular rule of the house being that the letters were to be put on the hall table, and then sent to the post. The point had been discussed just as if all the people in lunatic asylums were sane; but he would ask, supposing the inmates of those establishments were real lunatics, whether the rule in question was not a proper one. There was no improper restraint put on the plaintiff. Two days after the plaintiff was admitted to the asylum, he was visited by his sister and her husband, Mr. Fisher, and Mr. Wainwright, the attorney. That was on the 7th of November, 1857, and after that the Commissioners attended from time to time. If he was detained there too long, whose fault was that? Was it owing to the apathy of his relations? or was it not because they were satisfied there were good reasons for keeping him there? In former times people had been improperly detained in lunatic asylums in this country, as they had been formerly imprisoned in the Bastille; but as the Bastille had been broken into and destroyed, so also in this country, he had no hesitation in saying that, if any man had friends, he could not be kept in an asylum one week, or one day, after his friends believed he had been restored and had become sane. The object, in this inquiry, had been to withdraw the attention of the jury from the real question at issue, and the learned counsel for the plaintiff appeared as if he had rather been addressing a legislative assembly on some proposed plan for the amendment of the law on the subject of lunatic asylums. Much had been said against private lunatic asylums. But, would any man prefer Bedlam? Let him recollect the disclosures which were formerly made in connexion with that establishment. Would any man prefer being placed in a cottage, under a keeper, a low-classed man, with a medical man only occasionally in attendance? How much superior to either was a private lunatic asylum, conducted, as those institutions were, in accordance with modern improvements in treatment. Dr. Conolly, against whom so much had been said, was the great advocate of those reforms,—it was he who had struggled for years to take off restraint from lunatics, and who had enabled them to enjoy the pleasures of society and the benefit of country air. He had emancipated the wretched slaves who were formerly confined in dungeons. The learned counsel then proceeded to comment on the evidence, with a view to show that when the plaintiff was received into the asylum on the 5th of November, 1857, he was in a state of insanity, and required to be confined and taken care of. It was admitted by all that at that time he was labouring under delusions. The certificates also proved that, and he contended the certificates had not been proved to be false. The learned counsel commented at some length on those certificates to show that at that time the plaintiff was labouring under insane delusions. One point was, that he made "charges of cruelty against others, which are false." That was proved by what the plaintiff himself admitted—viz., that he had charged Miss Jones with putting her two children out of the way. Then he charged Mr. Barnett and his wife with drugging his wine. He charged his wife with infidelity. Who could tell whether those things indicated insanity better than the medical men who saw and talked with him? They also were the proper persons to judge whether he talked about his quarries like a madman or like a commercial man. It was not for Dr. Stilwell, the defendant, to call witnesses to prove the truth of the certificates; but for the plaintiff, if he could, to disprove them. Had he done so? He had not dared to call one witness to prove that he had not indulged in habits of intemperance. The truth of the certificates was proved by Mr. Ruck himself, for he admitted that when he thought he had had ocular demonstration of his wife's infidelity he asked her to go and live with him in Kent. It was proved that he kept loaded firearms, and that he took a loaded gun and pistols with him to Reading. All those statements contained in the certificates Dr. Stilwell was bound to take for granted, and to act upon, and even now they had not been proved to be untrue. The learned counsel also read Mrs. Ruck's order for the admission of the plaintiff into the defendant's asylum, and contended that, if her statement that his insanity was "probably partly hereditary" was unfounded, some member of his family beside the plaintiff ought to have been called to prove it so. If the plaintiff was not insane when he was sent to the asylum, why did not Mr. and Mrs. Fisher, and Mr. Wainwright, who saw him two days afterwards, do something towards his removal? Mr. Ackworth, his attorney, saw the plaintiff in March, but he did nothing. It was not till the 28th of April that Mr. Wainwright took any steps in the matter, and he then took down Dr. Seymour, who was not called as a witness. The learned counsel thought that a man who had delusions about his wife's fidelity, and who believed that his wine had been drugged by her paramour, was a dangerous lunatic, and common prudence required that he should be put under restraint, lest he should do harm to himself or to others. It was a fortunate thing that when the plaintiff got Mr. Barnett down on the ground two policemen came up and separated him from the man who he believed had been too familiar with his wife, and drugged his wine. Those same delusions continued all the time he was at the asylum, down to the 26th of July, 1858, when they all vanished at the bidding of the attorney, Mr. Wainwright, and he became satisfied that he was mistaken. The inquisition had lasted five days, and though a majority of the jury had found that he was not insane, there were grounds for believing that they might have been mistaken. The learned counsel contended that Dr. Stilwell had done everything in his power to cure his patient of his delusions. It was true he did not give him medicine more than once, for the medicine to be successful in such cases did not enter by the mouth; nor did he endeavour to persuade him that he was labouring under delusions, for that would have made him worse. But he administered the best of all medicines for an alienated and suffering mind, he gave him air and exercise, he allowed him commerce with the external world in excursions to Windsor and other places, and permitted him to attend the service at the village church on a Sunday. What physic could be better for a suffering mind than that? He was debarred from the use of spirits, but wine was allowed in the establishment. He had all necessary medical attendance, and was visited by the Lunacy Commissioners, who knew all about his treatment, and might have been called by the plaintiff as his witnesses. The plaintiff's case rested on the eloquent speech of his counsel, Mr. James, and his attack on Dr. Conolly. It was a very important question for the public to know, whether they were to be debarred from consulting that gentleman, in emergencies like these, merely because he happened to be the head of a lunatic establishment like that of Hanwell. The certificate which Dr. Conolly had signed had no reference to the defendant's establishment. It was a general certificate of the plaintiff's unsoundness of mind, and might have been produced at any establishment. It was said Dr. Conolly was a regular medical attendant at the defendant's house; but that was not the case. The book produced contained forty names, but Dr. Conolly only attended a portion of them.

Mr. Justice Hill.—The number varies from fifteen to eighteen.

Mr. Chambers said that the fact that the number varied showed that he was not a regular medical attendant, but only a consulting physician. Dr. Stilwell himself was the regular medical attendant, and his relative, Dr. James Stilwell, of Uxbridge. The payments made to Dr. Conolly did not make him a partner. It might as well be said that a barrister who received a retaining fee from the Bank of England or any commercial firm was a partner in the establishment, a proposition which was rather startling. Neither would his being paid by quarterly payments instead of by fees make Dr. Conolly a regular medical attendant. It was suggested that Dr. Conolly had acted from mercenary motives, and that because he received 700*l.* a-year from the establishment he would disgrace himself, and destroy his reputation by dishonourable acts. He (Mr. Chambers) treated that charge with the indignation it deserved; and he trusted the jury would not allow his honour to be attacked and his prospects blasted by such an imputation. It was not contrary to law for Dr. Conolly, as consulting physician, to sign such a certificate, so as to render himself guilty of a misdemeanour, which would subject him to three years' imprisonment, with hard labour. It was difficult when dealing with such a question to keep one's temper, though it was necessary for him (Mr. Chambers) to do so to the end. If Dr. Stilwell had made any misrepresentations respecting the plaintiff to Mrs. Ruck she might have been called as a witness. If any one was to blame in this matter, it was not the defendant, but Mr. and Mrs. Fisher, and Mr. Ackworth, and other members of the plaintiff's family, who ought to have interfered for his protection, and ought to have come forward now to disprove the charge of insanity. The learned counsel contended that the plaintiff had been taught to conceal the suspicions which he entertained of his wife. What had been his conduct since the commission? Mr. Jones tried to reconcile him to his wife, but he refused to be reconciled. Had he taken any notice of his six children? Had he seen or written to one of them? No. Yet he had not said one word against the kindness of Dr. Stilwell. The question, then, was, whether the defendant (Dr. Stilwell) was to blame, either when he received the plaintiff or when he detained him? If there had been any fault in Dr. Stilwell's treatment of him, the plaintiff might have sued him in an action for that neglect; but there was no foundation for such a charge, and it would not have stood the slightest examination. The very existence of the defendant now rested on the result of the verdict. The friends of the plaintiff were of opinion that he had done his duty to him, and he (Mr. Chambers) prayed the jury not to ruin his reputation and destroy his prospects, for by doing that they would prevent men of respectability from undertaking the delicate charge of watching over gentlemen in the plaintiff's condition, and would throw those duties into the hands of men of an inferior class, and into more careless hands. He would leave the defendant's case in the hands of the jury, and feel that it was safe.

The learned counsel's address, which was delivered with great earnestness, was followed by some applause.

Mr. Justice Hill then summed up the evidence to the jury. His Lordship said the action was brought by the plaintiff to recover damages from the defendant for imprisoning him among lunatics from the 5th of November, 1857, to the 27th of August, 1858. The defendant said he was justified in what he did; and the question for the jury to determine was, whether he was justified. It would be their duty to dismiss from their minds a vast deal of what they had heard discussed respecting the policy of the law. That was a question neither for the jury nor the judge, whose duty was to administer the law as they found it. One thing, however, was clear—viz., that most elaborate provisions had been made by the law for the protection of those who were placed in the unfortunate position of being confined in lunatic asylums. All human laws, however, were imperfect, but their defects must be remedied by the Legislature. A great change had been made in modern times in the mode in which lunatics were treated. Harshness and bodily restraint had given way to gentleness and soothing kindness, and the absence of bodily violence; and one gentleman, Dr. Conolly, had been proved to have taken a prominent part in bringing about this amelioration. But the question now was, whether the plaintiff's detention was justified by law. The defendant was the licensed keeper of a licensed asylum, and he said the patient was brought to him with a written order for his reception, and accompanied by two certificates, signed by two medical men, in the form pointed out by the statute, and that he was justified and bound to take charge of him till he died, or was discharged in due course of law. The plaintiff said there was a provision in the statute which forbade a certificate to be signed by certain parties; that Dr. Conolly's certificate was in violation of the Act of Parliament; and that the certificate was illegal, and no justification. It would be a question for the Court, whether the certificate, though sufficient in point of form, was sufficient in point of law. The provision was, "that no physician, surgeon, or apothecary who, or whose father, brother, son, partner, or assistant, is wholly or partly the proprietor of, or a regular professional attendant in, a licensed house, or hospital, shall sign a certificate for the reception of a patient into such house or hospital," &c. (16 and 17 Victoria, cap. 96, sec. 12.) The question which he (Mr. Justice Hill) should leave to the jury would be, whether they were of opinion, upon the evidence, that Dr. Conolly was "partly the proprietor of, or a regular professional attendant in, Moorcroft House; and, if so, he should direct them to find a verdict for the plaintiff for such damages as they might be advised. If, on the other hand, they should be of opinion that he was neither, he (Mr. Justice Hill) should direct them to find a verdict for the defendant. There would also be two other questions, whether Dr. Conolly examined the plaintiff separately and apart from Mr. Barnett, and also whether Mr. Barnett was in actual practice as a surgeon. His Lordship proceeded to observe, that there could be no doubt that the plaintiff had been suffering from *delirium tremens*, and he (the plaintiff) said that Dr.

Conolly and Mr. Barnett were both present all the time he was being examined, but there was no other evidence on that point, and the question would be, whether the jury could rely on the plaintiff's evidence in contradiction of the certificates, which stated that the parties had examined him "separately from any other medical practitioner." The next question would be, whether Dr. Stilwell kept the plaintiff *bonâ fide*, or whether he kept him there for his own gain, and those questions would be very important in considering the question of damages. His Lordship then read the plaintiff's evidence as to his treatment by the defendant, which showed that his delusions continued up to the 26th of July, 1858, and also the evidence of the keepers and Mr. Wainwright on the same point. His Lordship then referred to the book kept at the defendant's establishment, from which it appeared that in the first quarter of the year 1857 Dr. Conolly had received 152*l*. 10*s*. from the defendant. His Lordship thought that if Dr. Conolly had been partly the proprietor he would have received something out of every patient; but the book showed that he only received payments in respect of a certain number. It looked more like the case of a man who was "a regular professional attendant in" the asylum. In the second quarter, ending June, 1857, Dr. Conolly received his consulting fee (25 guineas) and payments in respect of eighteen out of forty patients. In the quarter ending at Michaelmas, 1857, Dr. Conolly received his consulting fee (25 guineas) and payments in respect of eighteen patients, in varying sums, amounting in all to 184*l*. 7*s*. 6*d*. In the quarter ending at Christmas, 1857, he received payments also in respect of eighteen patients. So, also, in the quarter ending in March, 1858, he received his consultation fee (25 guineas) and payments in respect of eighteen patients, and in that quarter Mr. Ruck's name appeared with 15*l*. opposite it. In the quarter ending at Midsummer, 1858, Dr. Conolly received his consultation fee of 25 guineas and payments in respect of eighteen patients, Mr. Ruck's name having 15*l*. opposite to it. It would be for the jury to say, upon that evidence, whether Dr. Conolly was or was not "a regular medical practitioner in" the asylum.

Mr. James called his Lordship's attention to the fact that while 15*l*. appeared twice after Mr. Ruck's name, Dr. Conolly had not attended him at all.

Mr. Justice Hill said that was certainly Mr. Ruck's evidence, but one of the witnesses said that Dr. Conolly spoke to him, and asked him how he did. His Lordship told the jury that, whether they thought Dr. Conolly was "in part a proprietor" or a "regular professional attendant" in the asylum, in either case they ought to find their verdict for the plaintiff, with such damages as they might think reasonable; but if they thought otherwise, they ought to find for the defendant.

The jury retired to consider their verdict, and on their return into court found that, if receiving the money as shown in the book made Dr. Conolly a part proprietor, they found the fact of receiving the money. They found that Dr. Conolly was the regular professional attendant, with 500*l*. damages.

As to Barnett's not being in practice, the jury found they had not sufficient evidence that he was not, nor had they sufficient evidence to satisfy them that the plaintiff had not been examined separately by Mr. Barnett and Dr. Conolly.

Mr. Justice Hill then directed the jury to find their verdict for the plaintiff, with 500*l*. damages, which was done accordingly.

Verdict for the plaintiff—Damages, 500*l*.

